# Problems of Control: Alcohol Dependence, Anorexia Nervosa, and the Flexible Interpretation of Mental Incapacity Tests

**DOI:** 10.1093/medlaw/fwy022

**Published:** 2018-07-23

**Authors:** Jillian Craigie, Ailsa Davies

**Affiliations:** 1 Centre of Medical Law and Ethics, Dickson Poon School of Law, King’s College London, Strand, London, UK; 2 Fitness to Practise, Nursing and Midwifery Council, London, UK

**Keywords:** Compulsion, Diminished responsibility, Mental capacity, Addiction

## Abstract

This article investigates the ability of mental incapacity tests to account for problems of control, through a study of the approach to alcohol dependence and a comparison with the approach to anorexia nervosa, in England and Wales. The focus is on two areas of law where questions of legal and mental capacity arise for people who are alcohol dependent: decisions about treatment for alcohol dependence and diminished responsibility for a killing. The mental incapacity tests used in these legal contexts are importantly different—one involves a ‘cognitive’ test, while the other includes an explicit impaired-control limb—and the comparison provides insight into a longstanding debate about the virtues of one type of test over the other. It is shown that both kinds of test can take control problems into account, but also that both can be interpreted in narrow and wide ways that significantly influence the outcome of the assessment. It is therefore argued that to a large extent, it is not the *kind* of mental incapacity test that matters, but how the test is interpreted. It is further proposed that value judgements are playing an unrecognised and inappropriate role in shaping this interpretation. This raises concerns about the current approach to assessing the impact of alcohol dependency on the capacity to make decisions about alcohol use or treatment, as well as broader concerns about flexibility within incapacity tests.

## I. SETTING THE SCENE: LEGAL CAPACITY AND IMPAIRED CONTROL

A long-recognised principle holds that mental incapacity can justify restricting a person’s legal rights or responsibilities, which together can be described using the term ‘legal capacity’.^1^ The legitimacy of linking legal capacity to mental capacity is currently under scrutiny in international human rights law and the conclusions of this article may be relevant to these unfolding debates.^2^ However, the focus of this article is law as it stands in England and Wales, where mental and legal capacity are closely linked. Paradigmatic examples are found in the Mental Capacity Act 2005 (MCA), which allows that self-determination in one’s personal affairs can be limited due to a mental incapacity; and the partial defence of diminished responsibility, which mitigates culpability on grounds of mental incapacity, reducing a charge of murder to manslaughter.

One issue that arises in both civil and criminal contexts concerns the ability of mental incapacity tests to account for control problems, which are often seen as central to addiction but also other conditions such as anorexia nervosa and compulsive hoarding.^3^ The literature has framed this issue as a question about whether so-called ‘cognitive’ or ‘rationality’ tests—which do not contain reference to impaired control—are sufficient, or whether such tests should contain an explicit impaired-control element. The test within the MCA is an example of a cognitive standard, referring only to the person’s ability to understand, retain, use and weigh information (and express a decision). In contrast, the law of diminished responsibility includes an impaired ability to ‘exercise self-control’ as one element within its statutory test.^4^

In relation to the capacity to consent to treatment, Louis Charland has argued that cognitive tests often fail to account for control problems.^5^ Using the example of addiction, Charland proposes that while associated impairments of appreciation and reasoning may be identified using such tests, compulsion is not straightforwardly accounted for. Similarly, with a focus on law in Victoria, Australia, Steve Matthews has argued that the mental incapacity required for involuntary treatment to be imposed for substance dependency, must be volitional rather than cognitive.^6^ In England and Wales, such concerns are related to the criticism that mental capacity law contains a cognitive or intellectual bias. However, these concerns have so far not focussed on addiction, though they have been raised in relation to anorexia nervosa.^7^

Parallel questions have been more extensively explored in the context of incapacity-based criminal defences. The issue in this context is whether cognitive tests for excuse or mitigation sufficiently allow problems of control to be taken into account. Among others, Michael Louis Corrado has argued that control problems should be considered as potential mitigating or excusing factors, and that cognitive tests are not sufficient in this regard. Corrado proposes that the incapacities relevant to criminal defences come in two distinct kinds. While incapacities to *grasp* one’s reasons can be identified using a cognitive test, incapacities to be *guided by* those reasons cannot.^8^

Against such views in the criminal context, Stephen Morse has argued that there is no persuasive conceptual account of control problems independent of cognitive problems. Moreover, he proposes that establishing whether a person has a control problem presents practical difficulties that go beyond those associated with identifying cognitive problems.^9^ According to Morse, it is theoretically within the scope of cognitive tests to identify the incapacities associated with substance dependence, for example, because such conditions can undermine the ability to:

‘think straight, to bring reason to bear on the reasons not to act. Some people in the throes of intense desires may be virtually unable to think of anything except satisfying the desire … Agents in such states will find it difficult to behave well because they have severe difficulty contemplating alternatives or coherently weighing alternatives. These are rationality problems.’^10^

‘If the craving sufficiently interferes with the addict’s ability to grasp and be guided by reason, then a classic irrationality problem arises and there is no need to resort to compulsion as the ground for excuse.’^11^

For Morse, control problems are rationality problems which can therefore be identified using a cognitive test. Morse suggests that insofar as cognitive tests for mitigation or excuse are failing to provide justice for people with a substance dependence, this is likely to be because the standards are too narrowly construed. The solution, in his view, is to broaden the scope of the cognitive tests.^12^ However, as Andrew Carroll and Andrew Forrester have pointed out, in practice this requires judges and juries to make an inference from impaired control to a cognitive incapacity.^13^ This raises a practical question about how straightforwardly addiction, for example, can be understood in terms of impaired abilities to understand or reason.^14^

This set of questions has been played out, among other contexts, in proposals for reform of the special verdict of not guilty by reason of insanity in various jurisdictions of the UK.^15^ While a number of common law jurisdictions include an explicit control element within a M’Naghten-based defence,^16^ UK jurisdictions have so far retained a cognitive version of this test. In 2004, the Scottish Law Commission reviewed this situation and recommended against the inclusion of an explicit control element. Instead it proposed that the M’Naghten criterion of a *failure to know* the nature or wrongness of one’s act should be broadened to a *failure to appreciate*. In the view of the Scottish Law Commission, and consistent with Morse’s position, a wide interpretation of *appreciate* would include control problems, making an explicit control element redundant.^17^

In 2013, the Law Commission of England and Wales considered the same issue and departed from the conclusions of the Scottish Law Commission, recommending the inclusion of a ‘lack of control limb’ as part of a new defence to replace the special verdict.^18^ The English proposal acknowledged the practical difficulties associated with a control limb in establishing whether a person’s capacity for control is impaired; as well as the argument that control problems are rationality problems and can therefore be accounted for within a cognitive test.^19^ However, it concluded, ‘we do not agree that it is always the case that irrational thinking and beliefs are all that lie behind a lack of control’, giving compulsive hoarding as a case in point.^20^

These debates highlight the possibility that the presence or absence of an explicit impaired-control element in the relevant standards may impact upon determinations of legal capacity in the context of alcohol dependence. This is an important diagnostic context in which to address these issues. The prevalence of alcohol dependence is estimated at around twice the prevalence of dependence on all illicit drugs, making it a serious social problem.^21^ It is also a complex problem because of the deeply embedded role for alcohol in UK culture. However, little attention has been paid to the way that alcohol dependence is viewed through mental incapacity tests in England and Wales, particularly in the civil context.

This article explores this issue with a focus on two areas of law: decisions concerning treatment for alcohol dependence, and responsibility for a killing. These are *prima facie* significant circumstances, and they are situations that arise for people who are alcohol dependent. However, the focus on these areas was largely predetermined by the fact that there are few contexts in which questions about the impact of alcohol dependence on legal capacity are raised.

The analysis begins with law concerning the capacity to refuse treatment for alcohol dependence, and a comparison with the approach to questions of the capacity to refuse treatment for anorexia. This comparison was chosen because of the significant case law in which impaired control is used to ground findings of mental incapacity to refuse treatment for anorexia. However, this comparison is made all the more relevant due to the case *A NHS Foundation Trust v Ms X,* which considers mental capacity in relation to both conditions.^22^

A debate about substance dependence and consent is then used to clarify the legal position in England and Wales concerning treatment refusal in relation to alcohol dependence and anorexia. The analysis of the anorexia cases shows that the MCA’s cognitive mental capacity test can be interpreted in a wide way that allows control problems to be taken into account; but suggests that a narrow interpretation of the test has been applied in questions concerning alcohol dependence.

The article then moves to the criminal law, tracing developments that have made the defence of diminished responsibility more available to alcohol dependent offenders. In contrast to the MCA, the incapacity test in this context does contain an impaired control limb, allowing a comparison of legal reasoning and outcomes in the presence or absence of an explicit control element. This analysis shows that the presence of a control limb does not automatically mean that the test is able to account for control problems. The mental incapacity test in the law of diminished responsibility was initially interpreted in a narrow way that made the defence unavailable to alcohol dependent offenders, despite its explicit reference to impaired self-control. Judicial developments have now widened its interpretation so that alcohol dependence can provide grounds for diminished responsibility.

These findings suggest that it is the interpretation of mental incapacity tests when they are applied, rather than whether they contain an explicit control element, that is crucial in determining outcomes in terms of legal capacity. The article concludes by arguing that value judgements associated with alcohol dependency and anorexia are playing a significant, unrecognised, and inappropriate role in driving the interpretation of mental incapacity tests.

## II. ALCOHOL DEPENDENCE AND THE CAPACITY TO REFUSE TREATMENT

Questions concerning an adult’s capacity to refuse treatment in England and Wales were for a long time decided using common law principles, but since 2007 they have been governed by the MCA. Since the implementation of the MCA, a person lacks mental capacity if, due to ‘an impairment of, or disturbance in the functioning of, the mind or brain’,^23^ they are unable to (i) understand, (ii) retain, or (iii) use or weigh relevant information in coming to a decision; or (iv) are unable to communicate their decision.^24^

Neither the MCA nor its Code of Practice discusses substance dependence. However, as Lady Hale has made clear, for the purposes of the Act a person ‘who is suffering the effects of alcohol or drug use or abuse, will be impaired or disturbed’.^25^ It would seem that alcohol dependence can therefore provide the basis for a finding of mental incapacity, if, for the Act’s purposes, the person’s decision-making abilities are relevantly impaired. However, research for this paper found only one reported case in which alcohol dependence was considered as potential grounds for mental incapacity.^26^ In *A NHS Foundation Trust v Ms X,* Ms X’s capacity to make decisions about alcohol use arose as a side issue to the central question of her capacity and best interests in relation to anorexia.^27^

Ms X was a young woman suffering from long-term anorexia and alcohol dependence.^28^ Due to an increasingly damaging cycle of imposed hospital admissions for anorexia, refeeding and weight gain, followed by excessive alcohol consumption and deliberate weight loss, the Trust sought a declaration that forced treatment for Ms X’s anorexia was no longer in her best interests and so would be unlawful.^29^

In its decision, the Court considered Ms X’s mental capacity both in relation to her eating disorder and her use of alcohol. Based on expert evidence, Cobb J was ‘entirely satisfied’ that Ms X lacked capacity in relation to decisions about her anorexia.^30^ However, the experts also agreed that Ms X *had* mental capacity in relation to her use of alcohol, and Cobb J accepted their opinion giving the following reasoning:

‘[The medical experts] both considered that Ms X was able to understand, retain, and crucially weigh up, the decision around drinking; they felt that her drinking was responsive to events – she appeared to be making choices about when to drink, when to drink more, and when to drink less. In particular, Dr. Glover was of the view that Ms X was able to weigh information such as the calorific content of alcohol, and appeared to be aware of the consequences for her liver functioning of continued abusive drinking, including the prospect that it could kill her.’^31^

It followed that the Court had no jurisdiction over Ms X’s decisions around alcohol consumption.^32^ The Mental Health Act 1983 (MHA) provides an alternative means by which an adult can be forcibly treated for a mental disorder, and this route does not depend on a finding of mental incapacity. However, drug and alcohol dependence are excluded from the MHA’s definition of a mental disorder, meaning that the statute does not apply directly to these conditions.^33^

This finding of mental capacity in the context of severe alcohol dependency raises a question about the MCA’s ability to account for this condition—at what point would alcohol dependency provide grounds for a finding of mental incapacity in relation to alcohol use?^34^ Further insight into this question comes from a recent case in which the impact of alcohol dependence on mental capacity was considered as a general issue. In *RB v Brighton & Hove City Council* the question of capacity concerned the impact of RB’s brain injury on his ability to decide about where to live.^35^ RB had been alcohol dependent since the age of 15—many years before his brain injury^36^—and following partial rehabilitation after the brain injury he wished to resume his prior way of life by moving into more independent accommodation; a decision that was predicted to result in a return to ‘alcoholism and a chaotic lifestyle’.^37^

On appeal, the decision that RB lacked the capacity to make this decision due to his brain injury was challenged on grounds that RB’s ‘inability to control his drinking is the same now as it was before the accident’.^38^ This argument relied on an assumption that RB had mental capacity in relation to his drinking prior to the injury; and therefore, that his alcohol dependency was unlikely to have undermined his capacity to make decisions in this area. While RB’s appeal was rejected, the Court affirmed the underlying assumption about alcohol dependence, holding that ‘an ordinary alcoholic … would not of course be made subject to a standard authorisation’.^39^ Given the context of the discussion it seems that ‘an ordinary alcoholic’ refers to an alcoholic without a brain injury (rather than commonplace alcohol dependency). This statement therefore appears to support the opinion of the medical expert, who gave evidence that, ‘Alcoholics can weigh up their decisions’.^40^

This position in relation to alcohol dependency stands in contrast to that adopted in several other common law jurisdictions. Australia^41^ and New Zealand,^42^ for example, have dedicated statutes that allow for involuntary detention and treatment on the basis of substance dependency, some of which require a loss of mental capacity in relation to the relevant substance.^43^ This difference in approach raises a question about whether the difficulties that people who are alcohol dependent can face in engaging with treatment are overlooked in English law; and whether this might be explained, in part, by a difficulty in accounting for control problems within the MCA’s cognitive test.^44^

An examination of law concerning refusals of treatment for anorexia provides insight into these questions, not only because of the juxtaposition of these two conditions in Ms X’s tragic case. A wider examination of relevant law shows that questions of mental capacity in this clinical context are often framed as questions about impaired control in relation to weight loss, and whether this results in a compulsion to refuse treatment. In the reported cases, it is often found that people with acute anorexia lack mental capacity to refuse treatment on these grounds.

## III. ANOREXIA NERVOSA AND THE CAPACITY TO REFUSE TREATMENT

A significant body of research supports the idea that anorexia can involve a severely compromised ability to control the restriction of nutrition and weight loss.^45^ Participants in studies by Jacinta Tan and colleagues reported significant difficulties in eating and accepting treatment even if they wanted to.^46^

In the case law both before and after the implementation of the MCA, impaired control is a central feature of anorexia held relevant to questions concerning the capacity make decisions about treatment. In *Re W*, the finding that W lacked the mental capacity to refuse treatment was based on the understanding that she had an ‘addictive illness’ which ‘creates a compulsion to refuse treatment or to accept treatment that is likely to be ineffective’.^47^ Similarly, E in *A LA v E* was found to lack capacity due to an ‘obsessive fear of weight gain’ that ‘overpowers all other thoughts’^48^; and in *B v Croydon HA* it was held relevant to the question of mental capacity that B was ‘unable to break out of the routine of punishing herself’.^49^ This approach to accounting for the impact of anorexia on the capacity to refuse treatment is now supported by the MCA’s Code of Practice, which states that,

‘a person with the eating disorder anorexia nervosa may understand information about the consequences of not eating. But, [they may nonetheless lack mental capacity because] their compulsion not to eat might be too strong for them to ignore’.^50^

Both prior to and since the implementation of the MCA these control problems in anorexia were described as impairing the person’s ability to deliberate about treatment. For example, during cross examination in *B v Croydon HA*, B apparently understood that her weight ‘was getting out of hand’, but she was held incapable of making an informed choice because the compulsion not to eat rendered her unable to ‘appreciate the extent to which she was hazarding her life’.^51^ The desire not to gain weight in anorexia was described as giving rise to ‘deranged thought processes’^52^ in *Re KB*, and to ‘distorting processes’ in *Re C*.^53^ Consistent with this approach, the MCA’s Code of Practice now advises that the compulsive features of anorexia can undermine a person’s ability to weigh information regarding the risks and benefits of treatment.^54^ This reasoning was applied in case *A LA v E*, when E was held able to understand, but unable to weigh the treatment information because of the overpowering nature of her need to not gain weight.^55^

In summary, the approach found in English mental capacity law concerning treatment for anorexia draws close associations between the concepts of addiction, compulsion, impaired deliberation, and mental incapacity. Descriptions of the experience of anorexia as being ‘stuck in the routine’ and ‘like a habit you can’t break’ are interpreted as evidence for mental incapacity;^56^ and such control problems are accounted for within the MCA’s cognitive test as an inability to weigh the risks and benefits of treatment.

These links drawn between addiction, compulsion, impaired deliberation, and mental incapacity show how the inference from impaired control to cognitive incapacity can, and is, being made within the MCA’s test. It suggests that an explicit control element is not necessary for taking impaired control into account within this structure. It also suggests that any difficulties associated with making such an inference do not play a central role in explaining the positions concerning alcohol dependency adopted in the cases of *Ms X* or *RB*. The MCA’s test could, it seems, be used to find that a person who is alcohol dependent lacks mental capacity in decisions about drinking and treatment.

In the medical ethics literature, Louis Charland has used arguments very like those found in the anorexia cases, to defend the view that heroin dependence undermines the capacity to consent in decisions involving heroin. His position shows how the argument for incapacity due to alcohol dependence might be made, and an analysis of this position clarifies what is at issue when questions of compulsion arise in cases concerning the refusal of treatment for anorexia. In the anorexia cases ‘compulsion’ refers to severe difficulties of control, not merely an absence of control. Behaviour can be willed—can involve a choice—and yet be compelled in the relevant legal sense.

## IV. HEROIN DEPENDENCE AND CONSENTING TO PRESCRIBED HEROIN

Charland argues that in the context of prescribing heroin as a treatment for dependency on the drug, the usual presumption in favour of capacity to consent should be reversed.^57^ Central to his case is the claim that compulsion is a defining feature of dependence, and that it undermines the ability to ‘weigh risks and benefits’ associated with heroin use.^58^ Charland concludes that for people who are heroin dependent, decision-making in connection with the drug is ‘warped’ and ‘biased’—in the case of a clinical trial involving heroin, the ‘benefits are overweighted’—and this casts serious doubt on the person’s ability to make these decisions.^59^

Charland’s reasoning is strikingly like that found in the English cases concerning refusal of treatment for anorexia. Applied to alcohol dependence in the context of English law, the argument would be that the compulsive features of this condition significantly warp or bias deliberation about drinking. Because treatment for alcohol dependence means not drinking—just as treatment for anorexia means not restricting nutrition—the alcohol dependent person’s ability to weigh the risks and benefits of treatment may be called into doubt.

Charland’s view on this issue has provoked criticism, among others, from Bennett Foddy and Julian Savulescu.^60^ They argue that a person is compelled only when the use of a substance is ‘irresistible’, removing choice, and in their view this is not an accurate description of heroin dependence.^61^ Among their supporting reasons, Foddy and Savulescu cite evidence that people who are heroin dependent can respond to strong incentives to not take the drug, and it is argued that they therefore retain a degree control. Understood in this way, even if a desire for heroin is extremely difficult to resist, use of the drug is always ‘volitional’^62^—it flows from the will rather than being a reflex or an epileptic seizure. Only a complete loss of control removes choice, rendering a behaviour irresistible, and so heroin use is not compelled.^63^

However, while Foddy and Savulescu’s interpretation of compulsion as a complete loss of control is one plausible view, it does not square with the use of this term in English law concerning the refusal of treatment for anorexia. Where compulsion is given as grounds for incapacity due to anorexia, it is most often described in terms of extreme distortions and biases in the decision process, rather than the person being deprived of a choice. In *Re C*, C was understood to be making a choice when it was said that, ‘worries about the effects on the body, and eventually threats to life itself, are ignored’.^64^ Similarly in *Re W*, compulsion in anorexia is said to involve ‘a firm wish not to be cured, or at least not to be cured unless and until the sufferer wishes to cure herself’, indicating that an anorexic person’s refusal of treatment is at least in part an expression of their current desires.^65^ Reflecting the understanding of compulsion found in these cases, the MCA’s Code of Practice advises that an anorexic person’s ‘compulsion not to eat might be too strong for them to ignore’.^66^ These descriptions clearly imply that the relevant decisions are volitions: they flow from the person’s beliefs and desires, even if their mental powers may be impaired. In these descriptions, the anorexic person is making a choice to refuse treatment yet this choice is compelled—a logical impossibility on Foddy and Savulescu’s view.

The understanding of compulsion in this part of English law is therefore wider than that proposed by Foddy and Savulescu in their analysis of heroin dependency. As a matter of English law, *severe difficulties* of control in anorexia, not merely an *absence* of control, can ground a finding of mental incapacity in relation to treatment. This understanding of compulsion has also been endorsed in the philosophical literature concerning addiction, with Jeanette Kennett arguing that compulsion is:

‘motivation that it is largely impervious, both to the agent’s values and to common techniques of self-control. This is consistent with the claim that the behaviour is intentional’. ^67^

Alcohol dependence could therefore involve compulsion in the relevant legal sense, resulting in a mental incapacity, even though drinking always involves a choice. The crucial legal issue in England and Wales is not whether the desire for alcohol is irresistible in the sense that drinking that is not willed, but rather the severity of the person’s difficulties in weighing the risks and benefits in decisions about treatment.

## V. REVISITING THE CASE OF Ms X

With this clarification in hand, we can now revisit the decision that Ms X had mental capacity in relation to her alcohol dependence. The justification given for this finding was that Ms X’s decisions about drinking responded to considerations other than her desire for alcohol, and that she therefore retained the ability to weigh in this area.

One interpretation of this reasoning is that it reflects the approach found in the anorexia cases. Understood in this way, Ms X’s weighing of other considerations was taken as evidence of a *sufficient degree* of responsiveness to reasons: Ms X retained enough control in her choices about drinking to retain legal capacity in relation to treatment. It is puzzling, however, that the main countervailing reason cited by the Court was the calorific content of alcohol. The experts and Court were firmly of the opinion that Ms X’s fear of weight gain resulted in a mental incapacity in relation to her anorexia. The fact that this same consideration played a role in her decisions about drinking does not therefore seem like good evidence that she retained sufficient control—understood as an ability to weigh—in relation to her drinking.

An alternative interpretation of the Court’s reasoning is that *any* responsiveness to considerations other than the desire for alcohol was taken to demonstrate Ms X’s ability to weigh in her decisions about drinking. So long as she demonstrated a choice to drink, the ability to weigh this decision was preserved, in line with Foddy and Savulescu’s narrow approach to the understanding of compulsion. The conclusions that can be drawn here are necessarily speculative. However, the expert opinion that Ms X ‘appeared to be making choices about when to drink’^68^ was accepted as evidence of her mental capacity in this area. This provides some evidence that a narrow approach was applied: a choice to drink meant that the ability to weigh was not relevantly impaired no matter what difficulties in resisting drinking, might have been involved.

If this second analysis is correct then the interpretation of the MCA’s test applied to Ms X’s alcohol dependence was markedly different to the interpretation applied in the anorexia cases. A question is also raised about whether this reflects the approach taken in wider practice, given that most mental capacity decisions don’t come to court. However, even if there is such a divergence of approach between these two diagnostic contexts, it might be argued that this is warranted. Two possible justifications are briefly considered.

One potential justification might be based on the claim that the stakes are higher in decisions concerning treatment for anorexia, and that this provides a reason to apply the mental incapacity test in a more stringent way. The underlying risk-relativity principle was part of common law prior to the MCA, but it is now endorsed only when the higher stakes mean the decision is more complex.^69^ The main problem for this form of justification concerns the underlying facts. There were 7,327 reported deaths in the UK in 2016 due to diseases that result directly from alcohol consumption, for example, alcoholic liver disease.^70^ The statistics clearly indicate a direct link between long-term alcohol abuse and death and in this sense the risks associated with alcohol dependence could not be higher. It may be that the predictability of death within a timeframe is more straightforward in malnutrition than in liver failure, but this seems a tenuous basis for a claim that the decision about treatment is therefore more complex.

Another potential justification might be based on a claim that forced treatment is unlikely to be in the alcohol dependent person’s best interests. Bernadette McSherry, for example, has noted a lack of evidence regarding the efficacy of civil commitment as a response to alcoholism.^71^ The question of mental capacity is independent of the person’s best interests in England and Wales. Nonetheless, there may be a reluctance to consider the question of mental capacity if a finding of incapacity would make no practical difference, because treatment would never be imposed. This line of thinking may play a part in explaining the small number of reported cases to consider the impact of alcohol dependence on the capacity to decide about treatment. However, serious concerns have also been raised about best interests in relation to forced treatment for anorexia. It has been argued that involuntary treatment can lead to a breakdown of trust with professionals^72^ and can result in chronicity that may make recovery less likely.^73^ The significant concerns in both clinical contexts suggest that this issue does not distinguish these conditions in a way that would justify adopting different approaches to the assessment of mental capacity.

In the absence of an obvious justification for the apparent difference in how the MCA’s test has been applied in the context of alcohol dependency and anorexia, the final sections of this article offer an explanation based on the very different value judgements associated with these two conditions. This proposal also raises wider concerns about the role that evaluative and other social inputs may be playing when the MCA’s mental capacity test is interpreted and applied in practice.

However, the article first examines how the impact of alcohol dependence on mental capacities is approached within the defence of diminished responsibility, using its test which contains an explicit control limb. The analysis traces an attempt in the criminal courts to make it possible for defendants to successfully plead diminished responsibility due to alcohol dependence. This has involved a shift in the understanding of ‘involuntary drinking’ from a narrow interpretation based on the absence of choice, to a wider interpretation referring to an impaired capacity for control. These developments engage with similar issues to those identified in the case of *Ms X*, illustrating that these difficulties are not avoided by the presence of an explicit impaired control element within an incapacity test.

## VI. DIMINISHED RESPONSIBILITY FOR A KILLING

Alcohol dependency is rarely considered as a potential mitigating or excusing factor for a criminal offence, though several commentators have called for changes to existing defences or the development of new defences, to allow substance dependency to be taken into account.^74^ The partial defence of diminished responsibility is the only place in English law where attempts have been made to use alcohol dependence *per se* to ground criminal mitigation or excuse.^75^

Over the past decade there has been an attempt to make this defence more available to alcohol dependent offenders, and these developments have addressed similar issues to those identified in the civil law above. The law of diminished responsibility was significantly changed in 2009. However, many of the important cases in relation to alcohol dependence pre-date those changes. Rather than providing an overview of developments in relation to diminished responsibility and alcohol dependency, this section of the article focusses on a crucial shift in how the impaired-control limb within the law of diminished responsibility has been interpreted.^76^

Prior to the 2009 amendments, the Homicide Act 1957 provided that a person who kills, or is party to a killing, should not be found guilty of murder,

‘if he was suffering from such abnormality of mind … as substantially impaired his mental responsibility for his acts and omissions in doing or being a party to the killing’.^77^

An abnormality of mind in this context included, ‘not only the perception of physical acts and matters, and the ability to form a rational judgment whether an act is right or wrong, but also the ability to exercise will-power to control physical acts in accordance with that rational judgment’.^78^

Among the 2009 amendments, an ‘abnormality of mind’ became an ‘abnormality of mental functioning’, although it was anticipated that the terms would not significantly differ^79^ and this has been confirmed in relation to alcohol dependency.^80^ The changes also modified the capacities relevant to mental responsibility by introducing three categories of ability—understanding, rational judgment, and self-control—at least one of which must be substantially impaired.^81^ As summarised in *R v Martin John Bunch*, the central issue for the alcohol dependent offender is now whether he or she,

‘was suffering from an abnormality of mental functioning which arose from that medical condition [alcohol dependency] and which substantially impaired one of the three capacities mentioned in the Act’.^82^

Crucially, the new statutory definition retains an explicit control element, although this was modified from an ability to ‘exercise will-power to control physical acts in accordance with that rational judgment’^83^ to an ability to ‘exercise self-control’.^84^ It is this element that has been used to argue for diminished responsibility on grounds of alcohol dependency. However, difficulties originally arose in defining and applying a test for a substantial impairment in this ability.

## VII. DIMINISHED RESPONSIBILITY AND INVOLUNTARY DRINKING

The case of *R v Tandy^85^* established that chronic alcoholism^86^ could be an abnormality of mind that substantially impaired mental responsibility for a killing. Tandy, a chronic alcoholic who strangled her daughter after consuming 90% of a bottle of vodka, was, however, unsuccessful in pleading diminished responsibility and convicted of murder.

The failure of the defence was due to Tandy’s inability to satisfy the Court’s test for whether her control in relation to drinking was relevantly impaired. In the absence of brain damage, which at that time was accepted as proof of an abnormality of mind,^87^ Tandy was required to prove that her consumption of alcohol prior to the killing was ‘involuntary’ due to her alcoholism,^88^ meaning she had ‘no immediate control’ over her consumption of alcohol.^89^ The craving for alcohol had to be irresistible in the sense that ‘the use of drink or drugs [was] involuntary’.^90^ Drawing a sharp distinction between a choice to drink and an abnormality of mind caused by disease, the Court of Appeal supported the trial judge’s direction that:

‘The choice [of whether to drink or not to drink on the day of the killing] may not have been easy but … if it was there at all it is fatal to this defence … . [If Tandy did drink] as a matter of choice, she cannot say in law or in common sense that the abnormality of mind which resulted was induced by disease’. ^91^

Applying this test for a substantial impairment of self-control,^92^ the jury were not persuaded that Tandy did not have a choice.^93^ However, *Tandy* raised the question of whether alcohol dependence could ever result in involuntary drinking in the required sense, and was heavily criticised on this basis. Julia Tolmie argued that the requirement of a total loss of control effectively took the defence of diminished responsibility away from people who are alcohol dependent.^94^ Similarly, Jonathon Goodliffe criticised the stark approach to assessing problems of control:

‘The Court of Appeal in *Tandy* were able to accept the adoption of diminished responsibility as it applies to alcoholism only in terms of black and white, rather than shades of grey: either the Defendant was wholly incapable of resisting the impulse to drink or she was responsible for her actions and should be convicted of murder.’^95^

In 2008 and 2009 the Court of Appeal took the opportunity to reassess *Tandy*^96^ in the cases of *R v Wood*^97^ and *R v Stewart*^98^ in which the defendants were diagnosed by all experts as suffering from alcohol dependency syndrome.^99^ In the trial of *Wood* the defence of diminished responsibility was rejected following the direction of the trail judge that, in accordance with *Tandy*:

‘A man’s act is involuntary if, and only if, he could not have acted otherwise. Giving into a craving is not an involuntary act, even if it is very difficult to do otherwise.’^100^

However, in Wood’s appeal the Court held that *Tandy* had imposed a strict test for a relevant impairment in the capacity for control, that, as in other prior cases,*^101^* was being very literally applied. The Court held that giving into a craving for alcohol could in fact constitute an involuntary act, confirming that, in law, an irresistible craving for alcohol can occur. According to this reassessment, a choice to drink in the context of alcohol dependency can be involuntary.^102^

There was also a shift of focus in *Wood*^103^ and *Stewart*^104^ away from the medical status of alcohol dependency. The critical issue in *Tandy* appears to have been whether Tandy suffered an ‘abnormality of mind … induced by disease’^105^ and it was to this issue that the question of involuntary drinking was applied.^106^ However, *Wood* confirmed the Court’s acceptance, by then, that alcohol dependency is ‘a true psychiatric condition’.^107^ This development raised a question about the role for the issue of involuntary drinking. The Court of Appeal set out that this issue is key both to the assessment of whether an individual suffered from an abnormality of mind resulting from alcohol dependency at the time of the killing;^108^ and if this element is made out, to the assessment of whether mental responsibility was substantially impaired.^109^ In *Stewart,* the Court of Appeal emphasised that, ‘[It] does not necessarily follow from the fact that a defendant suffers from alcohol dependency syndrome that he has established the necessary abnormality of mind’.^110^

## VIII. COMPLEXITIES AND FURTHER DEVELOPMENTS IN THE INVOLUNTARY DRINKING TEST

Stewart’s trial, however, foreshadowed challenges in the implementation of the revised test for involuntary drinking. In keeping with the reassessment set out in *Wood*, the defence expert concluded that Stewart’s ‘compulsion to drink was very great’, his ‘appetite for alcohol was overpowering’, and that ‘he had lost the ability voluntarily to control/resist a drink’.^111^ The prosecution expert, however, gave evidence that, ‘however chronic the alcoholism, an alcoholic always had a choice whether to drink’, that ‘alcohol dependence was not a disease in the typical sense like pneumonia, a condition over which the sufferer had no control’, and that, ‘[rather] than a disease alcoholism was a habit’.^112^ The Court of Appeal refrained from commenting on the expert’s opinion,^113^ but confirmed that, in law, an irresistible craving can occur in alcohol dependency.

Similar difficulties appear to have arisen in the more recent trial of *R v Williams*^114^ where an unused report by a medical expert was said by the Court of Appeal to rely on the approach in *Tandy*.^115^ However, a recent case indicates that there may be a growing acceptance in practice, of the revised understanding of involuntary drinking. In *R v Richardson*^116^ the Crown accepted a guilty plea of diminished responsibility due to alcohol dependency. The sentence was challenged by the Solicitor-General and was found by the Court of Appeal to be ‘unduly lenient’,^117^ but it was held that the Crown’s acceptance of the plea was nonetheless ‘entirely proper’.^118^

One emerging theme from the reported cases post-*Wood* and *Stewart* is an apparent shift in relation to the issue of an abnormality of mental functioning. In *R v Barry*,^119^ at trial and on appeal it was accepted that Barry was suffering from alcohol dependency at the time of the killing, and that this amounted to an abnormality of mind.^120^ In support of this position, the Court of Appeal outlined the prosecution’s expert evidence concerning the defendant’s ‘strong compulsion’ to drink, and his ‘difficulty in controlling’ his use of alcohol as well as withdrawal symptoms and tolerance to the effects of alcohol.^121^ However, the expert ‘was unable to say whether the appellant’s drinking had been voluntary or involuntary on the night in question’ and the defence’s medical expert report was said to have nothing to add.^122^ Contrary to the approach outlined in *Stewart*,^123^ it appears that in *Barry* the issue of involuntary drinking was not used to determine whether the defendant’s alcohol dependency amounted to an abnormality of mind.

Further insight is provided by two other recent cases. In *R v Williams,* the Court of Appeal indicated that an abnormality of mental functioning followed straightforwardly from a diagnosis of alcohol dependency. According to the Court, Williams ‘was suffering from an abnormality of mind, that is to say Alcohol Dependency Syndrome’,^124^ with the issue of voluntariness not raised until the discussion of Williams’ mental responsibility. In *R v Ashby,*^125^ the trial judge directed that the jury should consider whether Ashby was suffering from alcohol dependency syndrome at the time of the killing, and if he was, ‘the jury were then asked to consider on the balance of probabilities the applicant’s responsibility for stabling [sic] the victim was substantially impaired by the abnormality of his mind’.^126^ These directions leave no room for the possibility that the defendant may have been suffering from alcohol dependency, but not an abnormality of mental functioning, and the Court of Appeal made no criticism of these directions.

These observations suggest that the issue of an abnormality of mental functioning is now being tied closely to a diagnosis of alcohol dependency.^127^ Concerns were previously raised that the 2009 amendments to the law of diminished responsibility would bring further complexity to attempts to take alcohol dependence into account. It was suggested that increasing the number of issues that could be disputed and which require medical expert opinion to resolve, is likely to result in more contested pleas, which juries are less likely to accept.^128^ In relation to these concerns, the reported cases since the amendments came into force only provide limited insight. However, the impression given is that the approach to the alcohol dependent offender has not significantly changed. The shift to a more medicalised understanding of abnormal mental functioning may even have simplified this element of the defence by replacing the more nebulous issue of involuntary drinking.^129^

Nonetheless, these cases provide no insight into whether these developments have translated into a greater willingness on the part of juries, to accept a plea of diminished responsibility on grounds of alcohol dependency. The voluntariness of the defendant’s drinking at the relevant time remains the crucial issue in relation to responsibility. In the reported cases, there have been problems with the experts’ implementation of the revised involuntary drinking test, and juries may well be encountering the same difficulties.

## IX. WHAT THEN IS THE LEGAL POSITION OF THE PERSON WHO IS ALCOHOL DEPENDENT?

For persons who are alcohol dependent in England and Wales, questions of legal capacity in relation to decisions about treatment and in relation to their responsibility for a killing, turn on the person’s ability to control their drinking. However, the standard used to make this determination is different in these two contexts. The crucial element of the MCA’s cognitive test is the person’s ability to weigh the risks and benefits of alcohol use and treatment. The crucial element of the law of diminished responsibility now concerns whether the person was substantially impaired in their ability to exercise self-control; which turns on whether the person’s drinking was involuntary due to an irresistible craving for alcohol.

As described above, recent years have seen a shift in the law of diminished responsibility. Prior to *Wood*, the involuntary drinking test required a complete loss of control over drinking at the relevant time, which was incompatible with a choice to drink. However, judicial developments shifted the question of involuntary drinking into a grey area that concerns degrees of control, making this defence available in practice on grounds of alcohol dependency.

This has brought the approach to problems of control in the law of diminished responsibility significantly into alignment with that found in cases concerning the capacity to refuse treatment for anorexia. Here impaired control can provide a basis for a finding of mental incapacity even though the person’s refusal of treatment was a choice. Despite the MCA’s test being cognitive, and the test within the law of diminished responsibility containing an explicit control element, both allow volitional impairments to be taken into account. In both contexts, this is made possible by a wide interpretation of the relevant test, which allows impaired control, not merely an absence of control, to impact upon legal capacity.

However, the available evidence leads us to suggest that this wide approach is not being used in treatment decisions about alcohol dependency.^130^ We found no evidence of alcohol dependency being accepted as grounds for mental incapacity in relation to decisions about drinking or treatment; and only little evidence of the issue being considered at all. In the case of *Ms X,* the justification for the finding of mental capacity made particular reference to the idea that Ms X was making choices about drinking, which suggests that a narrow interpretation of the MCA’s test was applied.

These observations suggest that people who are alcohol dependent are in the following position. Their legal capacity is likely to be fully recognised in the context of decisions about treatment for alcohol dependency, even if their alcohol use is life-threatening. At the same point in time, if the person kills, their dependency may provide grounds for the partial withholding of legal capacity in relation to the killing, through a finding of diminished responsibility. The above analysis suggests this situation is sustained by a narrower interpretation of the relevant incapacity test in decisions about treatment for alcohol dependency.^131^ This narrow interpretation of the MCA’s test also contrasts with the wider interpretation of the same test in questions concerning treatment for anorexia, which has enabled findings of mental incapacity in this diagnostic context.

This situation raises a question about whether the difficulties that people with alcohol dependency may face in making decisions about treatment are being properly considered. It also raises a question about the desirability of the apparent flexibility within mental incapacity tests; and the drivers that are determining which interpretation will be applied in a particular context.

## X. NORMATIVE ANALYSIS: DIFFERENCES BETWEEN LEGAL CONTEXTS

Divergences in approach to mental and legal capacity across legal and clinical contexts are not necessarily problematic. Section V considered two possible justifications for adopting different approaches to applying the MCA’s incapacity test in the context of anorexia and alcohol dependency. The article returns to this issue later in this section, but it begins by considering some potential justifications for adopting different approaches to mental incapacity across the contexts of treatment decisions and diminished responsibility for a killing.

One important difference between the two legal contexts under consideration is that responsibility is only partially withheld in the defence of diminished responsibility. There is no equivalent outcome afforded within the MCA—for example, involving supported decision-making.^132^ In the absence of a middle ground within the MCA, the implementation of its test in a way that errs on the side of preserving legal capacity seems fitting with its guiding principles, which to some degree prioritise non-interference over the protection of well-being.^133^

However, this way of justifying the narrow interpretation of the MCA’s test in the context of alcohol dependency, is called into question by the anorexia cases.^134^ The wide approach to control problems in this diagnostic context has enabled interference in treatment decisions about anorexia, undermining any general appeal to the value of non-interference as a way of justifying the approach to alcohol dependency in decisions about alcohol use and treatment. In the absence of a plausible justification for applying different interpretations of the MCA’s ‘ability to weigh’ criterion in these two diagnostic contexts, there are broadly three ways to resolve this tension.

(1) One is to conclude that the incapacity test being applied in decisions about alcohol use is unduly narrow, perhaps making it impossible for someone to lack mental capacity in this diagnostic context. The narrow approach to control problems applied in the criminal case of *Tandy* provides a useful comparison, showing how in theory a finding of mental incapacity might be possible, without this being the case in practice. Based on the above analysis, we suspect that this is currently the case when the MCA is applied to questions of alcohol dependency and alcohol use or treatment.

As a remedy, lawmakers could bring the approach to assessing mental capacity in relation to alcohol dependency into line with that seen in the cases concerning treatment for anorexia. This would also bring into alignment the approach to assessing mental abilities in alcohol dependency across the treatment decision and diminished responsibility contexts. However, against this solution it might be argued that there are good reasons to adopt a narrower approach in the treatment decision context than in the law of diminished responsibility.^135^ It was argued above that non-interference is the most prominent value reflected in the MCA’s guiding principles, and that this provides a reason to interpret its incapacity test in a narrow way, prioritising the recognition of legal capacity. But what are the central values in law concerning a killing? Here, significant weight will be given to the value of the lives of others, and this consideration also pushes in the direction of recognising legal capacity, to enable blame and punishment.^136^ However, criminal law also places weight on the value of freedom, as reflected, for example, in the presumption of innocence in criminal trials.^137^ This consideration pushes in the opposite direction, towards a finding of diminished responsibility when responsibility is in doubt. If these two considerations are equally weighty, this suggests that a wider approach to control problems may be appropriate in the law of diminished responsibility than in the MCA.

(2) This points to an alternative solution, suggesting that the interpretation of the incapacity test applied in the anorexia cases may have been inappropriately wide, enabling interference where this was not warranted.^138^ Rather, on this thinking, the approach seen in relation to treatment for alcohol dependency better reflects the values of the MCA, and the normative differences between the civil and criminal contexts. The solution would be to narrow the interpretation of the incapacity test applied in treatment decisions about anorexia, bringing the approach in this context into alignment with that seen when the MCA is applied to questions of alcohol dependency, alcohol use, and treatment.

(3) A third response holds that there is truth in both the first and second responses, and calls for a reconsideration of practice in light of these concerns. This is the remedy we endorse. The above analysis suggests that insufficient consideration is being given to the difficulties that people who are alcohol dependent can face in relation to decisions about use and treatment. However, it also seems plausible that the interpretation of the MCA’s test in the anorexia cases has been inappropriately wide, too easily enabling interference in this diagnostic context. As one part of understanding how this situation may have come to be, and how it might be addressed, it is argued below that value judgements have played an inappropriate role in shaping the interpretation of the MCA’s test, when it is applied.

## XI. NORMATIVE DRIVERS IN MENTAL INCAPACITY ASSESSMENT

Evaluative considerations play an important part in shaping tests for mental incapacity. If a society strongly values individual freedom over personal well-being, then a capacity test concerning personal matters within that society is likely to place only minimal constraints on the abilities necessary to make one’s own decisions. In this way, values play a crucial part in establishing mental incapacity standards.^139^ In the addiction and socio-legal literatures it has also been observed that perceptions of addiction, shaped by historical context, have influenced how society responds.^140^ This observation raises the possibility that evaluative or other social factors are shaping the interpretation of incapacity tests in an unrecognised and unjustified way. It is argued here that such inputs play a significant role in explaining the divergent findings in relation to the MCA’s application in the context of alcohol dependence and anorexia.

Evidence of these inputs can be seen in Lord Donaldson’s judgment in *Re W*, a case concerning refusal of treatment for anorexia, when he draws a distinction between anorexia and other addictions:

‘Anorexia is an illness that is not the fault of the sufferer. In this it is no different from pneumonia or appendicitis … . It is an addictive illness although, unlike other addictions such as drug taking, the sufferer is not to be blamed for having allowed herself to become addicted.’^141^

The question raised by this position concerns *why* we should blame people with a substance dependency for their condition, but not those with anorexia for theirs. One explanation, suggested by the comparison of anorexia with pneumonia and appendicitis, is about the degree of involvement one has in developing these conditions. In most cases, a person plays little to no causal role in the development of pneumonia or appendicitis and this is the reason that we don’t blame them for getting ill. The problem with this explanation when applied to anorexia, is that the development of anorexia seems to require much more involvement on the part of the person. Like those who are alcohol dependent, people with anorexia play a significant part in the development of their condition, when they pursue diet and exercise regimes that at some point get out of control. In this way, anorexia looks much more like substance dependency than pneumonia or appendicitis.

On the other hand, what strongly distinguishes substance dependency from anorexia is that anorexia develops in the pursuit of things that our society values. Thinness and exercise are considered virtuous, along with traits associated with anorexia such as perfectionism and resistance against bodily desires. It is proposed here that these positively evaluated factors, associated with anorexia but not substance dependency, lie behind the traditional pattern of blame expressed in Lord Donaldson’s judgment.

A significant part of the concern here is that the motivation for the distinction drawn between substance dependency and anorexia in *Re W* is obscured. The explanation offered is that anorexia is like pneumonia and appendicitis, but the reasons for withholding blame in relation to these conditions are not the same. In the case of anorexia the reasons are primarily evaluative, while for pneumonia and appendicitis the reasons are primarily causal. The reference to pneumonia and appendicitis therefore conceals the motivating reasons for the assigning of blame in the case of substance dependency but not anorexia, making it seem that the distinction is much less value-based that it is.^142^

It also may be the case that stereotypes associated with substance dependency and anorexia play a role in traditional patterns of blame. The mental picture of a person with anorexia is likely to be a well-mannered young woman; while the mental picture associated with alcohol dependency is likely to be a disheveled older man. The work of Daniel Kahneman and colleagues suggests that the stereotypes evoked may well influence judgements about blame.^143^

It is proposed here that such evaluative and other social inputs—for example, gender stereotypes—play a significant role in explaining the differences observed between the approach to assessing mental capacity in the context of alcohol dependency and anorexia. Any number of psychological accounts could be given to explain how this occurs, but one possibility is sketched here to illustrate the idea.

Perhaps negative associations with alcohol dependency evoke feelings of anger and antipathy, and these connect to seeing the person as blameworthy. In assessments of mental capacity, the agency of the person—the target of the blame—may therefore be more salient than the impact of the impairment on decisions, making a narrow interpretation of the MCA’s test seem appropriate to apply. On the other hand, perhaps the positive associations with anorexia evoke feelings of sadness and sympathy, which connect to seeing the person as an innocent victim. This seems likely to foreground the impact of the impairment on decisions, making a wide interpretation of the MCA’s test seem appropriate to apply—enabling a finding of incapacity.^144^

Whether this illustration captures the psychology of what happens in practice, what’s clear is that the evaluative factors identified in this section should not be influencing the interpretation of the MCA’s incapacity test. Knowledge about whether a brain injury resulted from a heroic act, dangerous driving or a freak accident, should not impact upon the assessment of the injured person’s ability to make their own treatment decisions. Likewise, there should be no difference in the way the MCA’s incapacity test is interpreted in the context of alcohol dependency and anorexia, merely based on value judgements associated with these conditions.^145^ This raises questions about the assessment of mental capacity that go beyond these diagnostic contexts. As such, those responsible for assessing mental capacity should always be mindful that the MCA’s test can be interpreted in wide and narrow ways; and that evaluative judgements, which might not be part of conscious awareness at the time of the assessment, may influence the interpretation that is applied.

This article began by outlining a debate about the virtues of cognitive tests verses those containing an explicit control element, and it concludes by returning to this issue.

## XII. COGNITIVE VERSES VOLITIONAL TESTS

The above analysis demonstrates that mental impairments central to alcohol dependency can only be taken into account when incapacity tests are interpreted in a wide way that includes impaired control, not merely an absence of control. While this kind of observation has been made before in relation to cognitive incapacity tests, we have illustrated that the same point applies to tests that contain an explicit control element. The test within the law of diminished responsibility, with its control limb, was initially interpreted in a narrow way that made the defence unavailable on a basis of alcohol dependency. The solution adopted by the Court of Appeal was to hold that giving into a craving—behaviour that is willed—can, in law, be involuntary.

However, this solution retains the terms *involuntary* drinking and *irresistible* craving to describe the incapacity that is necessary for the defence, and we wonder whether this language is likely to lead experts and juries back to narrow, binary thinking about control. A less stark way of describing the control element of the test might mitigate against this problem. The MCA’s ‘use and weigh’ limb seems to offer an advantage on this count. When control is the central issue, questions of the capacity to refuse treatment for anorexia are discussed using language referring to distorted, biased, and overpowered thought. This makes it clearer that the question is not a binary matter about whether the person has any control at all, but one about the difficulties the person faces in relation to control.

This more subtle language, however, was not used in the judgments concerning alcohol dependency, with significance in the case of *Ms X* instead given to the idea that she was always making a choice to drink. When the impact of alcohol dependency on a person’s mental capacity was considered at all in the reported cases, it seems that a narrow interpretation of the MCA’s test was applied. We have argued that these findings are explained, at least in part, by certain kinds of evaluative or other social judgements playing an unrecognised and inappropriate role in the interpretation of the incapacity test, when it is applied. The upshot is that one of Morse’s central criticisms of explicit control elements, equally applies to cognitive tests. For Morse, the concern is that control elements lead to:

‘misleading metaphorical thinking about mechanisms and expert testimony that is little more than moral judgment wrapped in the white coat of allegedly scientific or clinical understanding’.^146^

If our analysis is borne out then this argument deployed against the inclusion of explicit control elements, in favour of cognitive tests, must be set aside. What appears to matter more than whether a test is cognitive or explicitly about control, is how the test is interpreted and applied. We propose that cognitive tests are just as vulnerable to interpretation that is inappropriately shaped by value judgements, as tests that include an explicit control element. These findings suggest that greater attention should be paid to way that value judgements can shape the interpretation of incapacity tests in practice, whether volitional or cognitive, and the concerning flexibility that can result.

## Footnotes

1 S Blumenthal, ‘The Default Legal Person’ (2007) 54 UCLA Law Review 1262; E Hasson, ‘Capacity to Marry: Law, Medicine and Conceptions of Insanity’ (2010) 23 Social History of Medicine 1.

2 P Bartlett, ‘The United Nations Convention on the Rights of Persons with Disabilities and Mental Health Law’ (2012) 75 Modern Law Review 752; A Ward and others, ‘Three Jurisdictions Report: Towards Compliance with CRPD Art. 12 in Capacity/Incapacity Legislation Across the UK’ (The Essex Autonomy Project, 2016).

3 In relation to addiction, impaired control in the use of the relevant substance is a defining feature within the major classification systems. Research into experts’ views about addiction has also shown that a loss of control, in some sense, is how addiction is most commonly defined. However, there is considerable disagreement about the extent to which control is impaired in addiction, and how this impairment should be understood. This is also evident in the legal analysis within sections VII and VIII of this article. A Carter and others, ‘Control and Responsibility in Addicted Individuals: What Do Addiction Neuroscientists and Clinicians Think?’ (2014) 7 Neuroethics 205; M Karasaki and others, ‘The Place of Volition in Addiction: Differing Approaches and their Implications for Policy and Service Provision’ (2013) 32 Drug and Alcohol Review 195; S Fraser, ‘Articulating Addiction in Alcohol and other Drug Policy: A Multiverse of Habits’ (2016) 31 International Journal of Drug Policy 6.

4 MCA s 3(1); Coroners and Justice Act 2009 s 52(1) A (c).

5 LC Charland, ‘Decision-making Capacity’ in EN Zalta (ed), *Stanford Encyclopedia of Philosophy* (2015); PS Appelbaum, ‘Ought we to Require Emotional Capacity as Part of Decisional Competence?' (1998) 8 Kennedy Institute of Ethics Journal 383.

6 S Matthews, ‘Addiction, Competence and Coercion’ (2014) 39 Journal of Philosophical Research 199. In a more recent piece, Matthews qualifies this claim by limiting it to cognitive incapacities in the here and now, as they are largely assessed, suggesting that cognitive capacities assessed over time may uncover relevant problems. S Matthews, ‘Addiction and Mandatory Treatment’ in H Pickard and S Ahmed (eds), *The Routledge Handbook of Philosophy and Science of Addiction* (Routledge, 2018).

7 J Tan and others, ‘Control and Compulsory Treatment in Anorexia Nervosa: The Views of Patients and Parents’ (2003) 26 International Journal of Law and Psychiatry 627. J Craigie, ‘Competence, Practical Rationality and What a Patient Values’ (2011) 25 Bioethics 326. For discussion of the ‘intellectual bias’ concern see: N Banner, ‘Unreasonable Reasons: Normative Judgements in the Assessment of Mental Capacity' (2012) 18 Journal of Evaluation in Clinical Practice 1038, 1040; T Gergel and G Owen, ‘Fluctuating Capacity and Advance Decision-making in Bipolar Affective Disorder: Self-binding Directives and Self-determination’ (2015) 40 International Journal of Law and Psychiatry 92, 93.

8 At least not without ‘redefining defects of reason to include defects of control’ ML Corrado, ‘Morse on Control Tests’ in PH Robinson, SP Garvey and KK Ferzan (eds), *Criminal Law Conversations* (OUP 2009) 461. In the same publication see also: SP Garvey, ‘The Folk Psychology of Self-control’ 460; SD Rozelle, ‘Sometimes a Control Test is just a Control Test’ 463; TA Maroney, ‘Why is a Folk-Psychological Account of Loss of Control Necessary (and what Precisely is it)?’ 465; RF Schopp, ‘Cognition, Rationality, and Responsibility’ 467; and A Gold, ‘Criminal Culpability and Self-control: Back to M'Naghten’ (2011) 18 Psychiatry, Psychology and Law 525.

9 SJ Morse, ‘Against Control Tests for Criminal Responsibility’ in Robinson, Garvey and Ferzan (eds), (n 8) 449; SJ Morse, ‘Hooked on Hype: Addiction and Responsibility’ (2000) 19 Law and Philosophy 3.

10 Morse, ‘Against Control Tests for Criminal Responsibility’, ibid 457–58.

11 Morse, ‘Hooked on Hype’ (n 9) 38.

12 ibid.

13 A Carroll and A Forrester, ‘Depressive Rage and Criminal Responsibility’ (2005) 12 Psychiatry, Psychology and Law 36.

14 ibid 39. Carroll and Forrester suggest that difficulties involved in making such inferences may be a significant barrier to defendants with control problems satisfying cognitive tests for mitigation or exculpation. They focus on a case concerning depressive rage in the Australian state of Victoria and the insanity defence in that jurisdiction.

15 For another example, in law concerning provocation: ML Baum, ‘The Monoaimine Oxidase A (MAOA) Genetic Predisposition to Impulsive Violence: Is it Relevant to Criminal Trials?’ (2013) 6 Neuroethics 287.

16 The English Law Commission gives examples in Ireland, Western Australia, and the US. Law Commission, *Criminal Liability: Insanity and Automatism, A Discussion Paper* (Crown Copyright 2013) 4.45.

17 Scottish Law Commission, *Report on Insanity and Diminished Responsibility* (The Stationery Office, 2004) 2.52–2.56. The Scottish report discusses the practical difficulties highlighted by Morse (2.53); and cited clinical opinion that no disorders are purely volitional with no cognitive impairment (2.54). However, it was noted that consultees were divided on the issue of whether the test should contain reference to volitional incapacities (2.54). Discussed in: Law Commission, *Insanity and Automatism Supplementary Material to the Scoping Paper* (Crown Copyright 2012) D.69–D.70.

18 It proposed that the grounds for the new defence should be that the ‘defendant wholly lacked the capacity: (i) rationally to form a judgment about the relevant conduct or circumstances; (ii) to understand the wrongfulness of what he or she is charged with having done; or (iii) to control his or her acts in relation to the relevant conduct or circumstances as a result of a qualifying recognised medical condition’. Law Commission (n 16) 1.93.

19 Law Commission (n 16) A.83–A.85.

20 ibid A.86; Further discussion: Law Commission, *Insanity and Automatism Scoping Paper* (Crown Copyright 2012) 2.41–2.43.

21 Farrell and colleagues found rates of alcohol dependence at 5% and illegal drug dependence at just over 2% overall, in a household survey within England and Wales. M Farrell and others, ‘Nicotine, Alcohol and Drug Dependence, and Psychiatric Comorbidity—Results of a National Household Survey’ (2003) 15 International Review of Psychiatry 50.

22
*A NHS Foundation Trust v Ms X* [2014] EWCOP 35.

23 s 2(1).

24 s 3(1).

25 B Hale, *Mental Health Law* (5th edn, Thomson Reuters 2010) 63.

26 This consisted of searches via the Westlaw database and consultation with experts in English mental health law. This article concerns alcohol dependency rather than intoxication or conditions that can arise from alcohol abuse, such as Korsakoff syndrome.

27
*Ms X* (n 22).

28 ibid 1. At the time of the hearing Ms X had been anorexic for 14 years and her alcoholism had resulted in end stage and irreversible liver disease.

29
*Ms X* (n 22) 6.

30 ibid 29. Both experts in the case held that Ms X’s false beliefs about her body and her fear of weight gain meant that she was unable to weigh information relevant to her eating disorder.

31 ibid 29.

32 ibid 20.

33 s 1(3). A person may be treated for a substance addiction under the MHA only if treatment of the dependency is aimed at treating a mental disorder, as defined by the Act, that is thought to arise from it. The exclusion of substance dependence from the MHA’s definition has been questioned on grounds that dependence is often responsive to treatment, and that being an alcoholic or drug addict is accepted as potentially legitimate grounds for detention in accordance with the European Convention on Human Rights art 5(1)(e). R Jones, *Mental Health Act Manual* (Sweet & Maxwell Ltd 2011).

34 It may have been right to allow Ms X to continue in her alcohol use without legal interference, based on her best interests. However, as the law stands, this should not have been a factor in the question of her capacity to make decisions in this area.

35
*RB v Brighton & Hove City Council* [2014] EWCA Civ 561.

36 ibid. RB was 37 at the time of the appeal hearing.

37 ibid para 87.

38 ibid para 33.

39 ibid para 25.

40 ibid para 25.

41 For a review of models in Australia: Mental Health Commission, *Compulsory Alcohol and other Drug Treatment in Western Australia* (2016, Government of Western Australia) 9–11.

42 Substance Addiction (Compulsory Assessment and Treatment) Act 2017.

43 For example, the Severe Substance Dependence Act 2010 (Victoria, Australia) only applies to persons who are ‘incapable of making decisions about his or her substance use and personal health, welfare and safety due primarily to the person’s dependency on the substance’. (s 5(c)). The Drug and Alcohol Treatment Act 2007 (New South Wales, Australia), does not include a loss of mental capacity in the assessment of whether involuntary detention and treatment is permitted. However, it does include restoring the person’s, ‘capacity to make decisions about their substance use and personal welfare’ as one of its aims (s 3(1)(d)). Within New Zealand’s Substance Addiction (Compulsory Assessment and Treatment) Act 2017, compulsory treatment is permitted only if, ‘the person’s capacity to make informed decisions about treatment for the addiction is severely impaired’ (s 7(b)).

44 For a discussion of this issue in a broader context: Matthews, ‘Addiction, Competence and Coercion’ (n 6).

45 George Szmukler and Digby Tantum argued that anorexia should be understood as an ‘addiction to starvation’. G Szmukler and D Tantam, ‘Anorexia Nervosa: Starvation Dependence' (1984) 57 British Journal of Medical Psychology 303, 303.

46 J Tan and others, ‘Competence to Make Treatment Decisions in Anorexia Nervosa: Thinking Processes and Values' (2006) 13 Philosophy, Psychiatry and Psychology 267; Tan and others, (n 7) 627.

47
*Re W (A Minor) (Medical Treatment: Court’s Jurisdiction)* [1993] Fam 64, 72, 81.

48
*A LA v E* [2012] EWHC 1639 (COP) para 49.

49 This case concerned whether force feeding amounted to treatment for mental disorder under the Mental Health Act but mental capacity was commented on, with the Court of Appeal finding that B lacked mental capacity. *B v Croydon HA* [1995] Fam 133, 141.

50 Mental Capacity Act 2005 Code of Practice 2007, s 4.22.

51
*B v Croydon HA* (n 49) 141.

52
*Re KB (Adult) (Mental Patient: Medical Treatment)* [1994] 19 BMLR 144, 145.

53
*Re C (A Minor) (Medical Treatment)* [1997] 3 FCR 49, 67.

54 MCA Code of Practice (n 50) s 4.21, 4.22.

55
*A LA v E* (n 48) para 49.

56
*B v Croydon HA* (n 49) 140.

57 LC Charland, ‘Cynthia’s Dilemma: Consenting to Heroin Prescription’ (2002) 2 American Journal of Bioethics 37.

58 ibid 41. His argument also relies on a related claim that ‘chronic heroin addiction results in radical changes in personal values that make seeking and using heroin the overriding goal of the addict’s life’. 41.

59 ibid 43.

60 B Foddy and J Savulescu, ‘Addiction and Autonomy: Can Addicted People Consent to the Presectipion of their Drug of Adiction?’ (2006) 20 Bioethics 1.

61 ibid 6, 7.

62 ibid 11.

63 Hanna Pickard is another theorist who adopts this understanding of compulsion, when she contrasts compulsion with duress. H Pickard, ‘The Purpose of Chronic Addiction’ (2012) 3 AJOB Neuroscience 40.

64
*Re C* (n 53) 67. We note, however, that it is also suggested that C had no control over the desire for the ‘gratification involved in being able to override pangs of hunger’ 67.

65
*Re W (*n 47) 72.

66 MCA Code of Practice (n 50) s 4.22.

67 J Kennett, ‘Addiction, Choice and Disease: How Voluntary is Action in Addiction?’ in N Vincent (ed), *Neuroscience and Legal Responsibility* (OUP 2013) 257.

68
*Ms X* (n 22) para 30.

69 Prior to the MCA the authority on this issue was Lord Donaldson in *Re T*: ‘What matters is that the doctors should consider whether at that time he had a capacity which was commensurate with the gravity of the decision which he purported to make. The more serious the decision, the greater the capacity required. If the patient had the requisite capacity, they are bound by his decision. If not, they are free to treat him in what they believe to be his best interests.’ *ReT (Adult: refusal of treatment)* [1993] Fam 95.

70 This figure does not include accidental or violent deaths where alcohol abuse may have been a factor (other than deaths due to alcohol poisoning) or diseases where alcohol is known to play a partial causal role such as in certain cancers. National Office of Statistics (2017) *Alcohol-specific deaths in the UK: registered in 2016*. https://www.ons.gov.uk/peoplepopulationandcommunity/healthandsocialcare/causesofdeath/bulletins/alcoholrelateddeathsintheunitedkingdom/registeredin2016

71 B McSherry and S Lenthall, ‘Laws to Detain Individuals with Substance Dependency: Breaching Human Rights or Restoring Health?’ (2011) 19 Journal of Law and Medicine 225; Stephen Morse also refers to the ‘undoubted but limited success of available treatment programs’, Morse, ‘Hooked on Hype’ (n 9) 23; More recent empirical data on the outcomes of compulsory treatment for substance dependency is discussed by Steve Matthews, who draws the conclusion that the results are uncertain: Matthews forthcoming (n 6).

72 J Tan, A Stewart and T Hope, *Treatment Decision-making in Anorexia Nervosa, an Empirical Ethics Research Validation and Dissemination Project* (The Ethox Centre 2008).

73 H Bruche, *The Golden Aage: The Enigma of Anorexia Nervosa* (Harvard University Press 1979) 97–99; T Carney, D.Tait and S.Touyz, 'Coercion is Coercion? Reflections on Clinical Trends in use of Compulsion in Treating Anorexia Nervosa’ (2007) 15 Australasian Psychiatry 390; S Giordano, 'Treating Eating Disorders: Some Legal and Ethical Issues’ in JRE Fox and K Goss (eds), *Eating and its Disorders* (John Wiley & Sons 2012) 102.

74 Morse, ‘Hooked on Hype’ (n 9); J Tolmie, ‘Alchoholism and Criminal Liability’ (2001) 63 Modern Law Review 688; G Yaffe, ‘Lowering the Bar for Addicts’ in J Poland and G Graham (eds), *Addiction and Responsibility* (MIT Press 2011) 178.

75 On the other hand, there is a significant literature on intoxication and criminal liability in England and Wales, for example: J Herring, C Regan and D Weinberg (eds), *Intoxication and Society: Problematic Pleasures of Drugs and Alcohol* (OUP 2012).

76 Coroners and Justice Act 2009, s 52(1). For a thorough analysis of these developments see: N Wake, ‘Recognising Acute Intoxication in Diminished Responsibility? A Comparative Analysis?’ (2012) 76 Journal of Criminal Law 71; M Gibson, ‘Intoxicants and Diminished Responsibility: The Impact of the Coroners and Justice Act 2009’ (2011) 12 Criminal law Review 909*;* RD Mackay, ‘The Coroners and Justice Act 2009 - Partial Defences to Murder (2) the Diminished Responsibility Plea’ (2010) 4 Criminal law Review 290.

77 Homicide Act 1957, s 2(1).

78
*R v Byrne* [1960] 2 QB, 396, 403.

79 Gibson (n 76)*;* Mackay (n 76).

80
*R v Adrian Joseph Barry* [2010] EWCA Crim 195; *R v Martin John Bunch* [2013] EWCA Crim 2498; *R v Williams (Dean)* [2013] EWCA Crim 2749, [2014] 1 Cr App R 23; *R v Ashby (Martyn)* [2015] EWCA Crim 712; *R v Richardson (James)* [2016] EWCA Crim 577.

81 The 2009 changes also introduced direct causal link between the alcohol dependency and the killing (Mackay (n 76)). This appears to go further than the House of Lords ruling in *Dietschmann,* which held that as long as the abnormality of mind existed at the time of the killing, and was a more than trivial cause of that act, it could be used to raise a plea of diminished responsibility (*R v Dietschmann* [2003] 1 AC 1209, para 18). Commentators raised concern that this may close down diminished responsibility for the alcohol dependent offender (Gibson (n 76)). However, we found no evidence that this has been the case.

82
*R v Martin John Bunch* [2013] EWCA Crim 2498, para 10.

83
*R v Byrne* (n 78) 403.

84 Coroners and Justice Act 2009, s 52(1A) (c).

85
*R v Tandy* [1989] 1 WLR 350.

86 In *Tandy,* this terminology was used rather than alcohol dependency syndrome, which was adopted in later cases.

87 This position was modified in *Wood* with the Court of Appeal holding that the involuntary drinking test should be applied whether or not the defendant had brain damage, although it was accepted that evidence of brain damage may make the case for an abnormality of mind more compelling. *R v Wood* [2008] EWCA Crim 1305, para 41. For discussion see: Wake (n 76).

88
*Tandy* (n 85) 356.

89 ibid 354.

90 ibid 357.

91 ibid 355.

92 ibid 354.

93 The Court of Appeal held the following to be sufficient evidence on which the jury could reach its verdict: The prosecution medical evidence that Tandy had the ability to abstain from taking the first drink on the day of the killing; Tandy’s own evidence that she had chosen to drink a stronger drink on the day of the killing, and that she might not have had a drink the day before; and that she had stopped before the bottle was empty. *Tandy* (n 85) 356.

94 Tolmie (n 74).

95 J Goodliffe, ‘R v Tandy and the Concept of Alcoholism as a Disease’ (1990) 53 The Modern Law Review 809, 810. As Gideon Yaffe has observed more broadly in the criminal context*:* ‘[I]f addiction does diminish responsibility it is not for the reason that, say, epilepsy diminishes responsibility. The epileptic might do damage when in the fit of a seizure, but she is not responsible for that damage since her spasmodic movements are not motivated. … But behavior stemming from addiction is not like this.’ G Yaffe, ‘Recent Work on Addiction and Responsible Agency’ (2002) 30 Philosophy & Public Affairs 178, 179.

96 In a general review of the partial defences to murder, the Law Commission recognised that there was a problem with the test for diminished responsibility adopted in *Tandy*, and it recommended that the issue should be resolved by judicial development. Law Commission, *Partial Defences to Murder,* report No 290 (2004), para 5.85.

97
*Wood* (n 87).

98
*R v Stewart* [2009] EWCA Crim 593.

99 In both cases a conviction of murder was quashed based on this reassessment. In *Wood* the Court of Appeal invited submissions on ‘whether a new trial should be ordered, or whether a conviction for manslaughter on the grounds of diminished responsibility should be entered’. *Wood* (n 87) para 43. In *Stewart*, a retrial before a jury was ordered (n 98) para 25. On retrial Stewart was convicted of murder, and a further appeal was dismissed. *R v Stewart* [2010] EWCA Crim 2159.

100
*Wood* (n 87) para 18.

101
*R v Inseal* [1992] Crim. L.R.35; *R v Atkinson* [1985] Crim. L.R. 314; *R v Egan* [1992] 95 CAR 278.

102
*Wood* (n 87) para 42.

103 ibid para 9.

104
*Stewart* (n 98).

105 As defined in s 2 of the Homicide Act 1957 prior to its amendment in 2009.

106
*Tandy* (n 85) 354.

107
*Wood* (n 87) para 9; *Stewart* (n 99) para 9.

108 ‘This depends on the jury’s findings about the nature and extent of the syndrome and whether, looking at the matter broadly, his consumption of alcohol before the killing is fairly to be regarded as the involuntary result of an irresistible craving for or compulsion to drink.’ *Stewart* (n 98) para 31.

109 In *Wood* the Court of Appeal was clear that in resolving this issue, ‘questions such as whether the defendant's craving for alcohol was or was not irresistible, and whether his consumption of alcohol in the period leading up to the killing was voluntary (and if so, to what extent) or was not voluntary’ are central. *Wood* (n 87) para 41.

110
*Stewart* (n 98) para 31.

111 ibid para 12.

112 ibid para 13.

113 ibid para 25.

114
*R v Williams (Dean)* [2013] EWCA Crim 2749.

115 ibid para 11. The Court of Appeal’s review of fresh evidence in this case found that in relation the question of mental responsibility, Williams’ ‘judgement and ability to control his impulses were seriously and significantly impaired’ due to alcohol dependency (ibid para 10), and a retrial was ordered.

116
*R v Richardson (James)* [2016] EWCA Crim 577.

117 ibid para 32.

118 ibid para 2.

119
*R v Barry* [2010] EWCA Crim 195. Barry was found guilty of murder and on appeal the conviction was found to be safe.

120 ibid para 3.

121 ibid para 10.

122 ibid para 11.

123
*Stewart* (n 98) para 33.

124
*Williams* (n 114). This opinion appears to have been based on expert evidence that his ‘ability to control his drinking was practically non-existent’ (n 114) para 10.

125
*R v Ashby (Martyn)* [2015] EWCA Crim 712, para 20.

126 ibid para 16. Ashby’s appeal was rejected on the basis that fresh evidence concerning alcohol dependency would not raise reasonable doubt as to his guilt.

127 For a discussion of a ‘recognised medical condition’ within the defence of diminished responsibility, particularly its relationship with diagnostic manuals, see *R v Dowds*, which concerned voluntary alcohol intoxication. *R v Dowds* [2012] EWCA Crim 281.

128 Mackay (n 76); Gibson (n 76).

129 It was always required that the abnormality arose from a medical condition. Prior to the 2009 changes, it was required that the abnormality arose, ‘from a condition of arrested or retarded development of mind or any inherent causes or induced by disease or injury’ (n 77) s 2(1); and following their implementation, ‘from a recognised medical condition’ (n 84) s (1)(a).

130 What happens in practice is unclear because the majority of decisions about mental capacity do not come to court, and until recently, of those that did, only a selection were reported.

131 The case of *RB* suggests that people who are alcohol dependent retain the ability to weigh in relation to their drinking (n 35); and the observation that Ms X made a choice to drink was accepted as evidence of her mental capacity in this area (n 22).

132 Either the person is judged to have mental capacity and their decision is respected; or they are deemed to lack mental capacity and a decision is made in their best interests.

133 This position is reflected, for example, in the principle that: ‘A person must be assumed to have capacity unless it is established that he lacks capacity.’ s 1(2); and that, ‘A person is not to be treated as unable to make a decision unless all practicable steps to help him to do so have been taken without success.’ s 1(3).

134 When control is perceived to be the central issue in this diagnostic context, a person can lack the ability to weigh the risks and benefits of treatment, despite having made a choice.

135 For a broader discussion of this issue: J Craigie, ‘Against a Singular Understanding of Legal Cpacity: Criminal Responsibility and the Convention on the Rights of Persons with Disabilities’ (2015) 40 International Journal of Law and Psychiatry 6.

136 Carroll and Forrester (n 13).

137 These are offered as just two key considerations, acknowledging that this is far from a complete analysis of the normative underpinnings of criminal law.

138 C Kong, ‘Beyond the Balancing Scales: The Importance of Prejudice and Dialogue in *A local authority v E and Others*’ (2014) 26 Child and Family Law Quarterly 216.

139 Camillia Kong has called for recognition of the role such factors play in best interests decisions within mental capacity law. Kong uses the hermeneutic concept of ‘prejudice’ to draw out the idea, where this refers not to a bias or negative prejudgement, but to the normative standpoint constituted by one’s historical context. ibid 222.

140 HG Levine, ‘The Discovery of Addiction: Changing Conceptions of Habitual Drunkenness in America’ (1978) 39 Journal of Studies on Alcohol 143; R Room, ‘The Cultural Framing of Addiction’ (2003) 6 Janus Head 221; D Courtwright, ‘The NIDA Brain Disease Paradigm: History, Resistance and Spinoffs’ (2010) 5 BioSocieties 137; S Fraser, ‘Junk: Overeating and Obesity and the Neuroscience of Addiction’ (2013) 21 Addiction Research & Theory 496; S Blumenthal, ‘The Default Legal Person’ (2007) 54 UCLA Law Review 1135, 1229, 1262; A Loughnan, *Manifest Madness: Mental Incapacity in Criminal Law* (OUP 2012), ch 7.

141
*Re W* (n 47) 73. A similar comparison was made by the prosecution expert witness in *Stewart* when they denied that alcohol dependence is a disease ‘in the typical sense like pneumonia’ (n 98) para 13.

142 Fabian Freyenhagen and Tom O’Shea have argued that mental capacity assessments often involve evaluative assumptions, for example in the context of anorexia, about the value of thinness. Their point is that claims about ‘distorted thinking’ in this context make assumptions about the value of thinness relative to other things such as life. This is an important observation. However, the point being made in the present article is that, whether or not the justification provided for a mental capacity decision involves assumptions of this kind, it may be shaped by unrecognised evaluative factors. F Freyenhagen and T O'Shea, 'Hidden Substance: Mental Disorder as a Challenge to Normatively Neutral Accounts of Autonomy’ (2013) 9 International Journal of Law and Psychiatry 53.

143 For example: D Kahneman and S Fredrick, ‘Representativeness Revisited: Attribute Substitution in Intuitive Judgment’ in T Gilovich, D Griffin and D Kahneman (eds), *Heuristics and Biases* (CUP 2002) 49.

144 This illustration of how evaluative judgements associated with diagnoses might influence mental capacity assessments, should not be taken to deny that alcohol dependency is recognised as a medical disorder. Alcohol dependency has been included in the major diagnostic standards for many decades, and the diminished responsibility cases discussed above demonstrate this status being recognised in law.

145 The fact that a dependency developed in pursuit of things that our society values (or not) does seem potentially relevant to the assigning of blame for resulting criminal acts. If someone developed a dependency while attempting to be a good citizen, then it seems reasonable to withhold blame to some degree, for negative consequences that follow. However, this should not be taken into account within a mental incapacity test, because this will obscure the justification for withholding blame.

146 Morse refers to tests that explicitly concern control as ‘the internal coercion or duress approach’. Morse, ‘Hooked on Hype’ (n 9) 38.

